# Biomimetic Research for Applications Addressing Technical Environmental Protection

**DOI:** 10.3390/biomimetics7040182

**Published:** 2022-10-28

**Authors:** Kirsten Wommer, Kristina Wanieck

**Affiliations:** Working Group Biomimetics, Faculty of Applied Informatics, Technology Campus Freyung, Deggendorf Institute of Technology (DIT), Grafenauer Str. 22, 94078 Freyung, Germany

**Keywords:** biomimetics, applied research, sustainability, innovation, biologically inspired design

## Abstract

Biomimetic research has increased over the last decades, and the development process has been systemized regarding its methods and tools. The aim of biomimetics is to solve practical problems of real-life scenarios. In this context, biomimetics can also address sustainability. To better understand how biomimetics research and development can achieve more sustainable solutions, five projects of applied research have been monitored and analyzed regarding biological models, abstracted biological principles, and the recognition of the applied efficiency strategies. In this manuscript, the way in which sustainability can be addressed is described, possibly serving as inspiration for other projects and topics. The results indicate that sustainability needs to be considered from the very beginning in biomimetic projects, and it can remain a focus during various phases of the development process.

## 1. Introduction

Modern society is often characterized by non-sustainable lifestyles and economies, as prominently shown by the measurement of the overshoot earth day (https://www.overshootday.org/newsroom/country-overshoot-days/; accessed on 9 May 2022). Therefore, substantial transformation in all areas of society is necessary to protect the environment and life on earth in general. Technology and innovation are vital fields of action which can address societal challenges. 

Biomimetics is considered to have a significant impact on science, technology, and society [1]. Its research has increased over the last decades, and the trend for an increasing number of publications is ongoing [1,2,3]. The topics covered by this research vary [1,4,5] showing the broad impact that biomimetics may have on various sectors and branches of society, from technology-oriented science and business innovation, to the economy at large [6,7]. Recent advances in the field deal with, among others, materials and surface technologies [8,9], fluid dynamics and robotics [10,11] and architecture [12].

Like biomimetics, sustainability is a very broad topic with many different perspectives. In biomimetics, biological strategies or principles, materials, structures, processes, and mechanisms can be used for new developments [13,14,15]. As natural systems show various strategies for material and energy efficiency, it is assumed that learning from biological models can lead to more sustainable solutions in the human-centered world, also called the “biomimetic promise,” [16,17]. Even though biology is not always sustainable per se and can also have negative environmental impacts, studies have shown that there are biological strategies and systems that can serve as models for sustainable biomimetic developments [18]. Several endeavors have been undertaken to link biomimetics to sustainability in theory [19,20], to address sustainable development in particular [21] as well as to assess and measure if and how biomimetic solutions contribute to sustainability and the various difficulties that may occur [22,23,24,25,26,27,28]. Such assessments vary in their origin, i.e., making a link to life cycle assessment (LCA), product sustainability assessment (PROSA), or developing new approaches, such as the bio-inspired sustainability assessment (BiSA; [24]) and other tools [29]. Some specific assessments dealt with biomimetic solutions, including façade painting inspired by the Lotus plant [26], a ceiling structure [22] or in architecture [24]. The results indicated that sustainability in biomimetic projects can be measured, but that a transfer of the used methodology to other projects and fields of development can be difficult. Therefore, more research is required to understand and analyze how biomimetics already addresses sustainability in development processes. 

One interesting aspect of biomimetics is that scientific-technological progress can be linked to sustainable and responsible innovation, as well as technical environmental protection, without using the biological model itself, as the biological model is not part of the solution [17]. In this manuscript, the focus is on environmental protection, which is defined by the United Nations (UN) as “any activity to maintain or restore the quality of environmental media through preventing the emission of pollutants or reducing the presence of polluting substances in environmental media” [30]. 

This might include the following effects of biomimetic developments:

- Direct and indirect saving of resources for the production and use of biomimetic products;
- Mitigation of environmental pollution during production;
- Energy savings in the production processes of biomimetic products;
- Energy savings in biomimetic applications;
- Reduced or easier recycling or disposal of products, or during their development process.

During 2019–2022, the Bavarian research association BayBionik–From Nature to Technology was coordinated by the authors of this paper. This research association aimed to develop biomimetic products and processes with the focus on biomimetics for technical environmental protection. In this context, the projects focused on “changes in characteristics of goods and services ” and “changes in production techniques,” as well as “recycling,” as specified in the above mentioned UN definition [30]. The aim was to successfully implement biological principles in technology which lead to (more) environmentally friendly or less ecologically harmful products and processes. BayBionik was financed by the Bavarian State Ministry of Environment and Consumer Protection, Germany, to develop sustainable and responsible innovation in research and development at academic institutions.

The authors of this paper supported six biomimetic research projects at five different universities in Germany. Table 1 summarizes the participating institutions with their respective projects. Next to the research projects, there was a seventh project focusing on outreach and scientific communication (P2). 

The activities of the coordination project included fostering cooperation and communication between the different research groups, identifying synergies as well as similarities or differences in the research approaches, supporting the research groups in addressing sustainability, exploiting the sustainability potential of biomimetics, and helping to overcome inter- and transdisciplinary challenges. Besides a specific insight into applied research projects, the authors intended to better understand whether and how the experience, results and findings of the research projects could be transferred to other projects/products, subject areas, or industries.

**Figure 1:**
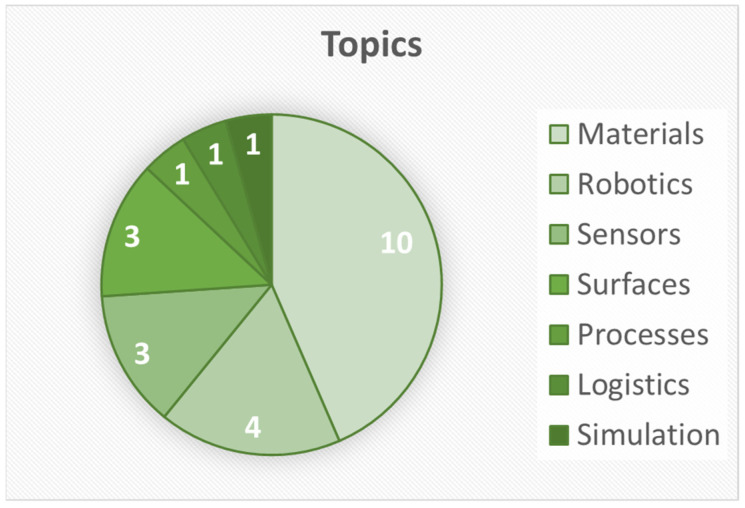
Topics of research proposals in the context of biomimetics addressing sustainability.

Before the project network started its work in 2019, a pre-study was conducted which identified research institutions in the context of biomimetics and asked them to apply for the funding opportunity of this association. To obtain funding, research institutions applied with a project idea, and a panel of four experts from the field of biomimetics examined the applications in a first screening. Figure 1 shows the topics of the initial project applications. 

A total of 23 institutions applied with research proposals, of which 6 were not considered to be biomimetic and were therefore excluded. Additionally, 3 project ideas did not fit into the program of the proposed network. The rubrics for evaluating the projects included scientific quality, connection to biomimetics and sustainability, focus on applications, and the potential for collaboration within the association. Only if the projects met more than half of the requirements were they then invited to present their work. As a result, the researchers of 14 projects were invited to present their ideas in a workshop, and finally, 6 research projects were chosen for funding. 

### Research Projects (P) 

Six of the eight projects of BayBionik were biomimetic research projects, covering two main topics, i.e., (1) self-cleaning sustainable surfaces (P3, P4), and (2) intelligent resource efficient systems (P5–P8). In the following, the projects are briefly described regarding the biological models used; the research idea, purpose, and process; as well as the results. Detailed information can be found in the respective scientific literature published regarding each project, as well as in the Results Section of this paper.

(1) Self-cleaning surfaces (P3)

Inspired by pitcher plants from the *Nepenthes* family, the aim of this project was to produce surfaces that avoid adhesion of, e.g., dirt, biofilms, or living organisms. With these new surfaces, the adhesion of mussels on ships, snails in agriculture, or icicles on gutters could be prevented. The project aimed to develop a simple, scalable, and sustainable coating process for liquid-repellent, self-cleaning, and non-fouling surfaces. An optimized spray-coating setup was developed leading to a reliable application of superhydrophobic coatings, with high homogeneity, in a single spray operation on glass substrates. In addition, the application method ensures a possible scale-up. The project developed a sustainable, scalable, two-step process for synthetically mimicking the pitcher plant that is based entirely on aqueous dispersions, thus minimizing the use of harmful organic solvents in the coating process [31,32].

(2) Sustainable surface functionalization (P4)

The surfaces of plastic products in daily use are exposed to environmental influences, dirt, and mechanical harm. To protect them, these products are often coated in an additional process step. However, the longer the products are in use and the more often they are cleaned, the more the coating is damaged, and its function is lost. 

The aim of project P4 was therefore to develop surfaces that can renew themselves. To this end, additives were selected that could migrate to the surface of a material independently, following nature’s example of migrating substances. These additives, i.e., silicones, were tested in various concentrations as possible functional additives and mixed into plastics (ABS and PMMA) to form an easy-to-clean surface. Silicone additives were selected, which fulfilled both a depot function and the premise of a strongly reduced surface energy. Further effects of the selected compound were an improved scratch resistance of PMMA and a reduction in the processing temperatures. The coating is required to work over a long period of time, and the additional process step of coating with possibly environmentally hazardous substances should be avoided. 

(3) Bio ceramics (P5)

The examples of bones, teeth, or mussel shells show that many biological models build up an efficient bio ceramic layer by layer. These materials are evolutionarily optimized for energy efficiency and performance and are produced at moderate temperatures. The layer-by-layer structure often gives them extraordinary properties, such as very high load-bearing capacity with low material usage.

The aim of the project was to establish a biologically inspired synthesis process for ceramics and to use an energy efficient and resource saving process. Such materials could be used for bone implants, for example, and in the long term, could represent a sustainable alternative to the energy-intensive ceramic processes. In this project, the newly developed biomimetic synthesis approach, a layer-by-layer process, is particularly suited to generate analogous crystallographic gradients and bioactive coatings for biomedical implants in an energy efficient synthesis [33,34,35].

(4) Optical fibers made of spider silk (P6) and cellulose (P7).

The project “BionOptik—Bionic high-tech materials for optical applications” comprises the two sub projects of the University of Bayreuth (P6; BionOptik I) and the Technical University of Munich (BionOptik; P7 II). The aim of the project was the production of biologically inspired “fiber optical cables” made of the biological materials cellulose and spider silk. The hierarchical structure of the fibers was inspired by *Euplectella aspergillum.*

Project P6 works with spider silk, which is believed to form the robust and flexible cover of the fiber due to its outstanding mechanical properties. For this purpose, a hybrid protein of spider silk and a cellulose binding domain could be produced, characterized, and successfully processed. Project P7 uses cellulose particles that are to act as optical conductors. This involves the production of novel optical fiber architectures from environmentally friendly, non-toxic, and biodegradable substances. Developed cellulose fibers were successfully coated with the spider silk protein without delamination and are thus suitable as biopolymer optical fibers [36,37].

(5) Robotic Owl Neck Joint (P8)

Conventional robots are often heavy and require a great deal of energy. Joints in robotics can be optimized, as inspired by biological models. For example, owls can turn their heads almost completely around their own axis. The exact analysis of this movement, particularly the uninterrupted blood supply of the brain during the strong rotation of the neck, are the focus of this project. The research team analyzed the interactions of the owls’ neck and used this information to design more energy-efficient joints for construction machinery or handling robots used in healthcare. As a result, a technical prototype was developed which uses actuators made of shape memory alloys [38].

The biomimetic design process has several challenges and limitations, and these might become pitfalls in transferring biological knowledge to engineering [39]. Therefore, one aim of the coordination of the BayBionik project network was to investigate how the biomimetic development process has been realized successfully in the respective projects and how the underlying principles of biological models led to specific applications. The challenges and drawbacks were also analyzed. Two projects of BayBionik (P3 and P4) exhibited a collaboration with partners from industry. Therefore, the projects were also analyzed regarding their inter- and transdisciplinary cooperation within the teams. To find out how the development process was realized, several methods were used.

## 2. Materials and Methods

The research projects were supported by the coordination project and supervised by the authors of this paper. The aim was to monitor the research and to understand how the biomimetic development process was applied in the research projects. The data was derived from the following resources:

(1) Each project published a final report (available at www.baybionik.de; accessed on 16 August 2022) which provided insight into the development processes. 

(2) An initial survey was answered by the projects’ team members in 2019 (n = 6). Another survey at the end of the project was completed by the team members (n = 6) and project leaders (n = 5) in 2022.

(3) In person visits to each research project, in the laboratories, were planned. Due to the COVID-19 pandemic, however, only two projects were visited for in-depth discussions (P4 and P8). All other meetings between March 2020 and March 2022 took place online, except for those during summer 2021, when in person meetings were again allowed. 

(4) Regular meetings and discussions during the project duration.

### Surveys

The surveys were designed to gain insight into four main topics: 1. The biological models; 2. The research and development process; 3. The technical environmental protection; and 4. Aspects of interdisciplinarity. Among others, the following main questions were monitored and analyzed.

Biological models:

- Which biological systems served as models for the applied research?
- On which hierarchical level were the biological systems analyzed and used?
- What was their level of abstraction?
- How much information about biology was necessary for the development of the application?
- Which biomimetic process (solution-based or problem-driven) was used?

Research:

- Was it a new development, or did you rely on previous work?
- Where did the knowledge about biology come from?
- Did any difficulties arise regarding the biomimetic development process?
- Which problem was solved with the new development?
- Which objectives regarding sustainability should be achieved?
- Did you identify target groups for the development?

Team composition and interdisciplinary cooperation:

- Were biologists involved in the projects?
- Was there an active cooperation with a biology institute?
- How many different disciplines were involved?
- Did the team members develop new skills? If yes, which ones?
- Did challenges arise due to the team composition?
- Was industry involved?

Technical environmental protection:

- How was technical environmental protection addressed?
- Was a product or a process developed?
- Which biological strategies regarding sustainability were used?
- Was the contribution to environmental protection assessed? If yes, how?

## 3. Results

### 3.1. Research Projects

#### 3.1.1. Biological Models and Their Abstracted Principles and Strategies

Each research project focused on one biological model for which applied research should take place. Table 2 summarizes the various biological models of the respective projects and their abstracted principles.

#### 3.1.2. Technical Environmental Protection

The research projects addressed various sustainability topics linked to biomimetics. Table 3 summarizes the strategies that could be linked to the respective projects.

Vincent et al. 2006 compared the ways in which technical problems are solved in engineering and in biology [40]. One example in this comparison is that in technology, materials and energy are usually used to solve a problem, e.g., for surface characteristics, while in biological systems, the problem is usually solved via structure. This fact can be seen with the examples of P3 and P4, in which two plant surface structures inspired the development of a self-cleaning surface. In P4, an additional layer on the technological surface was avoided, reducing the use of environmentally harmful substances. As seen from this example, the contribution of biological models to sustainable development might not be obvious at first glance, and a deep understanding of the product requirements and the aim of the product at the very beginning of the biomimetic development process are necessary.

#### 3.1.3. Inter- and Transdisciplinary Characteristics and Challenges

**Figure 2:**
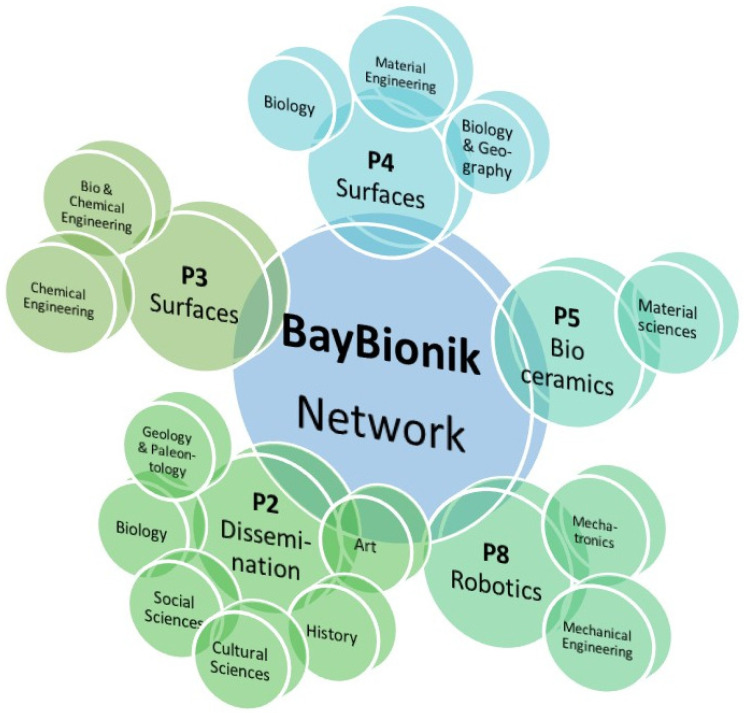
Scientific disciplines of the team members involved in the projects. Project leaders added their expertise. P6 and P7 did not offer detailed information. P2 was added, as it showcases the variety of disciplines that can contribute to biomimetic projects.

Most teams involved two to four members of various disciplines. Figure 2 shows the disciplines involved and their connection to the projects. In this manuscript, interdisciplinary means the involvement of various scientific disciplines. Transdisciplinary means the cooperation between academic and non-academic partners, i.e., partners from industry or society. 

Projects P3 and P4 involved collaboration with a partner from industry, i.e., with a brand for designing protective gear (P3), and with the automotive sector (P4).

Projects P3, P4, and P5 included biologists or related biological expertise on the team. P8 used members with extensive experience in performing biomimetics projects in engineering and accessed in-depth knowledge of biology from external partners, i.e., another university with fundamental research activities in biology, as well as the Zoo of Nuremberg. Therefore, they stated that no internal biology expertise was needed.

All project leaders stated that the interdisciplinary communication and exchange, as well as the different perspectives, were very helpful and well established in the research groups. 

#### 3.1.4. Assessment of Environmental Protection

The research projects aimed to develop products and/or processes that address sustainability. In all projects, the technical feasibility had a higher priority than addressing technical environmental protection, which means that first, the biological principle needed to be successfully transferred to technology, leading to an application with the defined properties. In a next step, the chances for achieving a positive sustainable impact were identified and applied. For the measurement of the sustainable impact, no clear and common method was used in all projects. Each project decided on its own at which steps in the biomimetic process substances could be substituted, whether energy could be saved, and if process steps could be optimized. 

P8 performed an LCA regarding the used a shape memory alloy. The LCA of P8 referred to the ISO standard on environmental management and LCA [41]. The shape memory alloys were compared to two other actuators, i.e., electric motors and pneumatic cylinders, regarding resource conservation and energy efficiency. The main categories that were compared were required resources, efficiency of the actuators, product lifetime, generated noise, and recycling rate. The evaluation showed that, despite the high energy requirement, the developed shape memory alloys are characterized by low resource consumption and high recycling potential [42].

#### 3.1.5. Developments—Products and Processes

Due to the COVID-19 pandemic, the research projects suffered from long periods of restricted access to universities and the respective laboratories. However, all teams were able to finish their projects in 2022, and their resulting products are summarized in Table 4. These developments were presented at a final meeting on 17 March 2022. All projects started from scratch, even though the concepts and potential applications were well-known in theory.

As mentioned earlier, P3 and P4 exhibited cooperation with partners from industry from the very beginning. The other projects needed to identify potential target groups and industry sectors which could be interested in the results. Once companies were identified, they were informed about the network. P4 was contacted by three other companies that showed interest in their developments. As a result, P3 and P4 will conduct a follow-up project with new partners from industry to improve their developments.

## 4. Discussion

Funding: Project funding in the field of biomimetics is still challenging, as many funding organizations do not include biomimetics [2]. The project association was funded directly by a state ministry, which is a privileged situation. The focus of this funding program was to support applied research that aims to inform the development of new biomimetic products or processes. The focus of this network was on the applied research, and no pure research could be funded. This aspect was mandatory during the application phase of this program. Projects that did not address this requirement could not be funded. In biomimetics, as described earlier, the valley of death between research and application, or even market-ready products, is challenging to overcome [43]. Having a funding opportunity that helps bridge this gap can enable biomimetic research to be more likely to deliver a prototype, a minimal viable product, or a new process.

Interdisciplinarity: The role of biologists in biomimetic project teams has been discussed previously [44,45,46], and the deep knowledge of biological models is a crucial part of the biomimetic process. On the other hand, the systemization of the process and the development of computational tools facilitating the process can enable engineers to complete the steps of the biomimetic process on their own [47,48,49]. This raises the question of team composition and different roles on biomimetic teams [45,50,51] as well as how much these participants need to be trained in the process and its tools [49,52]. The team composition of the research projects described here shows that three research projects included biological expertise on their teams (P3, P4, P5), and one team relied on external biological expertise (P8). None of the projects mentioned that any interdisciplinary challenges arose from within the teams. This is an interesting observation, and it shows that their research worked well, based on the transferable skills of team members. The experience of P8 shows that pure engineering institutions can also successfully develop biomimetics products, and that biological knowledge can be accessed via external cooperation. This highlights again how important a fundamental knowledge of biology is for biomimetics. It will be interesting to see how the work of institutions that focus on fundamental biological knowledge will be used in biomimetics research in the future, as well as how this cooperation can be fostered.

The interdisciplinary character of biomimetic projects leads to several challenges in practice, as indicated by projects in the context of industrial applications [43,53,54,55]. During the research projects presented in this paper, such challenges were limited, as the team members were familiar with interdisciplinary cooperation. This raises the question of whether interdisciplinary challenges arise are more likely to arise in the industrial context, where academic and non-academic participants need to communicate and cooperate. The two projects that involved partners from industry (P3 and P4) did not state any challenges regarding the cooperation with industry. It was mentioned that there were regular meetings, a good collaboration, and a high interest from the industrial partners (P3). P4 stated that towards the end of the project, collaboration could have been more effective, which might have been limited due to pandemic restrictions. Both projects involved the industrial partner from the very beginning, as they were part of the final application for the grant. This could be one important aspect, as no other inducement of industry was needed, their commitment was documented as part of the proposal, and they could express their needs and capabilities very early in the process.

Research and development process: All research groups had already identified a biological model when starting their projects. Therefore, the solution-based process was primarily performed, as well-known biological principles or strategies were transferred into a technical application. However, the initial motivation to perform these projects was the desire to solve a practical problem, i.e., addressing technical environmental protection in the context of a biomimetic application. The projects of BayBionik show that the two processes have overlapping phases, and in practice, they are not always easy to differentiate clearly. 

Each research project focused on one biological model, except for P6/P7, in which both cellulose and technical spider silk were used for technical optical fibers. Additionally, P4 followed a second approach, which encompassed the problem-driven approach by defining various technical needs (e.g., migration of additives, transport of substances, surface formation) for which additional biological models were researched. As such, the solution-driven approach was supported by additional inspiration from biological models that could make the first idea of application even better.

P3 (*Nepenthes* surface) shows that once the transfer and application of a biological principle to technology works in the lab, various fields of application can be identified, as seen in Table 4. During this project, the contribution of the industrial partners was important, as they identified new fields of application once the application did not function for glass surfaces, as initially planned. This fact emphasizes that there is a mutual learning in biomimetics, i.e., during the process, more information about the biological system and the technological requirements is acquired once the first prototypes have been developed.

P4 (self-cleaning surface) benefited from its collaboration with industry and the production of a real product, i.e., caravans are currently in field testing under real-life scenarios. Additionally, the transfer to other fields of application is under investigation, both with the initial industry partner and its portfolio and with three other companies that have attended the final presentation of the project network and showed their interest.

P5 (bio ceramics) was able to develop a functional process, i.e., layer by layer assembly under mild conditions, that is faster than those of compared processes. The material is as robust as other materials regarding its stability and fracture strength. 

The results of P6 (spider silk)/P7 (cellulose) show that the initial idea of biomimetic research does not necessarily lead to a successful implementation, as the process phase of abstraction (step 6) and transfer to technology (step 7) require in-depth knowledge of both biology and technology. The in-depth knowledge in biology was provided by the extensive experience of the two research groups. The transfer to technology and the performance of the resulting application were the goal of the research projects. The reason why the combination of spider silk and cellulose did not work out as expected was mainly due to different material qualities (e.g., spider silk led to opacity) or different incompatible solving agents during the production of both materials, which could not be overcome with variations of the processes. However, new ideas for application appeared, e.g., in medical devices where a transparent material is not necessary, but the biocompatibility of spider silk is crucial. The results of P6/P7 also show that new fields of application can be identified once a process works in the lab or once a product’s specificity is known.

Project P8 led to an energy efficient robotic arm with a high rotation flexibility. 3D printing allowed to produce a lightweight structure, providing material resource efficiency. After three years, a market-ready product is not yet available, but the first version clearly shows what is needed for improvement.

Link to environmental protection and sustainability: Assessing the contribution of biomimetics to sustainability varies from one development to the other. The question remains regarding whether it is possible to design a general assessment methodology for any biomimetics project. The ISO 18458 states that it was impossible to describe a general biomimetics process, as projects vary too much [17]. The same could be true for the contribution of biomimetics to sustainability, as the topics, the impact, and the processes might vary too much. Table 3 shows that each project could identify a clear contribution to different aspects of sustainability, and they all stated that other methodologies could have been used. However, there is no clear description of how to use existing methodologies in each biomimetic project, even though it is possible to characterize one’s contribution, as described by Speck et al. 2017 [25]. Topics such as the substitution of certain material or chemical agents used or other ways of saving resources could be addressed more easily; however, there must be a willingness of the projects to consider these topics. Moreover, team members must be aware and motivated to address sustainability, which has already been examined in industrial contexts [56]. In all research projects presented here, technical feasibility was deemed to be more important than sustainability impact. This is reasonable, as the new process or functionality first needs to work in the technical context. However, this raises the question of when, how, and to what extent sustainability should be addressed in biomimetic projects. 

## 5. Conclusions

The research projects described in this paper show that each biomimetic development is unique regarding its motivation, how the biomimetic process is used, how much biological knowledge is necessary, which phases of the biomimetics process are more challenging than others, and the effectiveness of cooperation with industrial partners. Once a process works well in the lab, or a well-known characteristic of a biological model can be transferred, suitable fields of application must be identified early so that the development is likely to solve problem on the market. However, new fields can be identified when new knowledge clarifies the actual needs of the technical design. Additionally, no clear methodology of how to address sustainability in general in biomimetic projects could be deducted; rather, each project must identify the potential impact on sustainability early on and cross-check its feasibility throughout the project. Various methods can help to assess the project’s contribution. Future research will focus on how to address sustainability in biomimetic research and development and how this focus can be facilitated by specific tools and methods. 

## Figures and Tables

**Table 1:** Institutions participating in BayBionik.

Institutions	Type	Project
Deggendorf Institute of Technology (DIT)	University of Applied Sciences	P1 Coordination
Bionicum	Educational Institution	P2 Scientific communication and outreach
Friedrich-Alexander- Universität Erlangen- Nürnberg (FAU)	University	P3
		Self-cleaning surfaces
DIT	University of Applied Sciences	P4 Sustainable surface functionalization
FAU	University	P5 Bio ceramics
University of Bayreuth	University	P6 BionOptik I
Technical University of Munich	University	P7 BionOptik II
Technical University Nuremberg	University of Applied Sciences	P8 Robotic owl

**Table 2:** Biological models and their abstracted principles for research application.

Biological Model (Research Project)	Observed Phenomenon	Abstracted Principle	Application	Reference
Nepenthes plant (P3)	Ants can walk on the edge of the flowers; at a certain moment, they slip off	Anti-adhesive surface	Self-organized surface with repellent functionalities; applications for self-cleaning, anti-adhesion, anti-ice	[31,32]
Lotus plant (P4)	Self-cleaning properties	Structured superhydrophobic surface	Surfaces with low surface energy; self-organized surface structure based on migrating additives	
Mussel shell (P5)	Strong and robust material; efficient material usage	High load bearing capacity	Bio-inspired ceramics based on a low energy process	[33,34,35]
Spider silk (P6)	Mechanical properties	Elastic and tear-resistant fibers; manage structural forces	Tubes made of spider silk proteins	
*Euplectella* *aspergillum* and cellulose (P7)	Optical fibers made of nano SiO_2_	Light transmitting properties	Biodegradable optical fibers made of cellulose (material architecture rebuilt with cellulose)	[36,37]
Owl (P8)	Turns head 270°	Rotation without clamping	Energy-efficient robotics kinematics	[38]

**Table 3:** Biological models and their sustainability strategies addressed in the respective research applications.

Biological Model (Research Project)	Environmentally Friendly Solvents	Sustainable Resources	Avoidance of Toxic Substances	Avoidance of Cleaning Agents	Dirt Repellence	Self-Cleaning Properties	Extension of Product Life Cycle	Process Optimization	Resource Efficiency (Material (M)/Energy I)	Improved Recycling/Biodegradable
Nepenthes plant (P3)	X	O	X	X	X	X	G	X	O	O
Lotus plant (P4)	O	O	O	X	X	X	G	O	O	O
Mussel shell (P5)	O	O	O	O	O	O	X	X	X (M and E)	O
Spider silk (P6)	O	X	X	O	O	O	O	O	X	X
Cellulose (P7)	O	X	O	O	O	O	O	O	X (M and E)	X
Owl neck (P8)	O	O	O	O	O	O	O	O	X (M and E)	O

X: Addressed; O: Not addressed; G: Goal.

**Table 4:** Developments of the research projects with potential fields of application.

Project	Developed Product or Process	Fields of Application
P3 Nepenthes	Surface coating process Dirt- and fluid-repellent surface on glass Anti-snail surface Anti-adhesion of mussels underwater Cement-repellant surface on shoes	Protection gear Glass surfaces Underwater application
P4 Lotus	Easy-to-clean surface on an automotive component Self-organizing surface structure	Automotive New fields are to be identified
P5 Bio ceramics	Production under mild conditions Biocompatible material	Medical application Implants
P6 Spider silk	Biodegradable robust material	Optical applications
P7 Cellulose	Light transmitting fibers	Optical applications
P8 Owl	Robotic prototype	Handling assistant Maintenance work

## Data Availability

Additional information regarding the research network can be found at www.baybionik.de (accessed on 27 October 2022).

## References

[1] Lepora N.F., Verschure P., Prescott T.J. (2013). The state of the art in biomimetics. Bioinspir. Biomim..

[2] Jacobs S., Wanieck K. (2022). Biom*: On Becoming a Teachable Discipline. Biomimicry for Materials, Design and Habitats.

[3] Lenau T.A., Metze A.-L., Hesselberg T. (2018). Paradigms for biologically inspired design. Bioinspiration, Biomimetics, and Bioreplication VIII.

[4] Gerbaud V., Leiser H., Beaugrand J., Cathala B., Molina-Jouve C., Gue A.M. (2022). Bibliometric survey and network analysis of biomimetics and nature inspiration in engineering science. Bioinspir. Biomim..

[5] Lurie-Luke E. (2014). Product and technology innovation: What can biomimicry inspire?. Biotechnol. Adv..

[6] Ferdinand J.P., Petschow U., Gleich A.V., Seipold P. (2012). Literaturstudie Bionik. Schr. IÖW.

[7] Mead T.L. (2014). Biologically-Inspired innovation in large companies: A path for corporate participation in biophysical systems?. Int. J. DNE.

[8] Feng S., Delannoy J., Malod A., Zheng H., Quéré D., Wang Z. (2020). Tip-induced flipping of droplets on Janus pillars: From local reconfiguration to global transport. Sci. Adv..

[9] Feng S., Zhu P., Zheng H., Zhan H., Chen C., Li J., Wang L., Yao X., Liu Y., Wang Z. (2021). Three-dimensional capillary ratchet-induced liquid directional steering. Science.

[10] Menzer A., Gong Y., Fish F.E., Dong H. (2022). Bio-Inspired Propulsion: Towards Understanding the Role of Pectoral Fin Kinematics in Manta-like Swimming. Biomimetics.

[11] Zeng H., Wang Y., Jiang T., Xia H., Gu X., Chen H. (2021). Recent progress of biomimetic motions-from microscopic micro/nanomotors to macroscopic actuators and soft robotics. RSC Adv..

[12] Cruz E., Hubert T., Chancoco G., Naim O., Chayaamor-Heil N., Cornette R., Menezo C., Badarnah L., Raskin K., Aujard F. (2021). Design processes and multi-regulation of biomimetic building skins: A comparative analysis. Energy Build..

[13] Graeff E., Maranzana N., Aoussat A. (2020). Biological Practices and Fields, Missing Pieces of the Biomimetics’ Methodological Puzzle. Biomimetics.

[14] Bhasin D., McAdams D. (2018). The Characterization of Biological Organization, Abstraction, and Novelty in Biomimetic Design. Designs.

[15] Fish F.E., Beneski J.T. (2014). Evolution and Bio-Inspired Design: Natural Limitations. Biologically Inspired Design.

[16] Gleich A., Pade C., Petschow U., Pissarskoi E. (2010). Potentials and Trends in Biomimetics.

[17] (2015). Biomimetics—Terminology, Concepts and Methodology.

[18] O’Rourke J., Seepersad C.C. (2015). Using Biology as A Model for Sustainability: Insights for Ecodesign and Bioinspired Design Practitioners.

[19] Helfman Cohen Y., Cohen Y.H., Reich Y. (2016). Biomimetic Design Method for Innovation and Sustainability.

[20] De Pauw I., Kandachar P., Karana E., Peck D., Wever R. (2010). Nature Inspired Design: Strategies Towards Sustainability.

[21] Kennedy E.B., Marting T.A. (2016). Biomimicry: Streamlining the Front End of Innovation for Environmentally Sustainable Products. Res.-Technol. Manag..

[22] Antony F., Grießhammer R., Speck T., Speck O. (2014). Sustainability assessment of a lightweight biomimetic ceiling structure. Bioinspir. Biomim..

[23] Horn R., Albrecht S., Haase W., Langer M., Schmeer D., Sobek W., Speck O., Leistner P. (2019). Bio-inspiration as a Concept for Sustainable Constructions Illustrated on Graded Concrete. J. Bionic. Eng..

[24] Horn R., Dahy H., Gantner J., Speck O., Leistner P. (2018). Bio-Inspired Sustainability Assessment for Building Product Development—Concept and Case Study. Sustainability.

[25] Speck O., Speck D., Horn R., Gantner J., Sedlbauer K.P. (2017). Biomimetic bio-inspired biomorph sustainable? An attempt to classify and clarify biology-derived technical developments. Bioinspir. Biomim..

[26] Antony F., Grießhammer R., Speck T., Speck O. (2016). The cleaner, the greener? Product sustainability assessment of the biomimetic façade paint Lotusan® in comparison to the conventional façade paint Jumbosil®. Beilstein J. Nanotechnol..

[27] de Pauw I.C., Kandachar P., Karana E. (2015). Assessing sustainability in nature-inspired design. Int. J. Sustain. Eng..

[28] O’Rourke J.M., Seepersad C.C. (2012). A Methodology for Identifying Factors That Contribute to the Sustainability of Bioinspired Engineered Systems. Proceedings of the ASME 2011 International Mechanical Engineering Congress and Exposition.

[29] Terrier P., Glaus M., Raufflet E. (2019). BiomiMETRIC Assistance Tool: A Quantitative Performance Tool for Biomimetic Design. Biomimetics.

[30] United Nations, Department for Economic and Social Information and Policy Analysis, Statistics Division Glossary of Environment Statistics, Studies in Methods. https://unstats.un.org/unsd/publication/SeriesF/SeriesF_67E.pdf.

[31] Chiera S., Koch V.M., Bleyer G., Walter T., Bittner C., Bachmann J., Vogel N. (2022). From Sticky to Slippery: Self-Functionalizing Lubricants for In Situ Fabrication of Liquid-Infused Surfaces. ACS Appl. Mater. Interfaces.

[32] Walter T., Hein T., Weichselgartner M., Wommer K., Aust M., Vogel N. (2022). Dispersion-based, scalable fabrication of repellent superhydrophobic and liquid-infused coatings under ambient conditions. Green Chem..

[33] Myszka B., Schodder P.I., Leupold S., Barr M.K.S., Hurle K., Schüßler M., Demmert B., Biggemann J., Fey T., Boccaccini A.R. (2020). Shape Matters: Crystal Morphology and Surface Topography Alter Bioactivity of Bioceramics in Simulated Body Fluid. Adv. Eng. Mater..

[34] Wallis D., Harris J., Böhm C.F., Wang D., Zavattieri P., Feldner P., Merle B., Pipich V., Hurle K., Leupold S. (2008). Biominerals with Texture Gradients are Functionally Graded Bioceramics Toughened by Stress Delocalization. arXiv.

[35] Wallis D., Harris J., Böhm C.F., Wang D., Zavattieri P., Feldner P., Merle B., Pipich V., Hurle K., Leupold S. (2022). Progressive changes in crystallographic textures of biominerals generate functionally graded ceramics. Mater. Adv..

[36] Reimer M., van Opdenbosch D., Zollfrank C. (2021). Fabrication of Cellulose-Based Biopolymer Optical Fibers and Their Theoretical Attenuation Limit. Biomacromolecules.

[37] Reimer M., Zollfrank C. (2021). Cellulose for Light Manipulation: Methods, Applications, and Prospects. Adv. Energy Mater..

[38] Löffler R., Rücker D., Müller F., Hornfeck R. (2021). Method for simulative reproduction, verification and technical adaptation as part of biological kinematics studies. Procedia CIRP.

[39] Wolff J.O., Wells D., Reid C.R., Blamires S.J. (2017). Clarity of objectives and working principles enhances the success of biomimetic programs. Bioinspir. Biomim..

[40] Vincent J.F.V., Bogatyreva O.A., Bogatyrev N.R., Bowyer A., Pahl A.-K. (2006). Biomimetics: Its practice and theory. J. R. Soc. Interface.

[41] (2020). Umweltmanagement_- Ökobilanz_- Anforderungen und Anleitungen (ISO_14044:2006_+ Amd_1:2017_+ Amd_2:2020); Deutsche Fassung EN_ISO_14044:2006_+ A1:2018_+ A2:2020.

[42] Hornfeck R. (2022). Ein Eulenhalsgelenk für Effizientere Maschinen: Abschlussbericht Teilprojekt Nr. https://baybionik.de/wp-content/uploads/2022/03/Abschlussbericht_P8_BayBionik_Eulenhalsgelenk-1.pdf.

[43] Chirazi J., Wanieck K., Fayemi P.-E., Zollfrank C., Jacobs S. (2019). What Do We Learn from Good Practices of Biologically Inspired Design in Innovation?. Appl. Sci..

[44] Graeff E., Maranzana N., Aoussat A. (2019). Biomimetics, where are the biologists?. J. Eng. Des..

[45] Graeff E., Maranzana N., Aoussat A. (2019). Engineers’ and Biologists’ Roles during Biomimetic Design Processes, Towards a Methodological Symbiosis. Proc. Int. Conf. Eng. Des..

[46] Snell-Rood E. (2016). Interdisciplinarity: Bring biologists into biomimetics. Nature.

[47] Wanieck K., Fayemi P.-E., Maranzana N., Zollfrank C., Jacobs S. (2017). Biomimetics and its tools. Bioinspired Biomim. Nanobiomater..

[48] McInerney S., Khakipoor B., Garner A., Houette T., Unsworth C., Rupp A., Weiner N., Vincent J., Nagel J., Niewiarowski P. (2018). E2BMO: Facilitating User Interaction with a BioMimetic Ontology via Semantic Translation and Interface Design. Designs.

[49] Pentelovitch N., Nagel J.K. (2022). Understanding the Use of Bio-Inspired Design Tools by Industry Professionals. Biomimetics.

[50] Wanieck K. (2022). Biomimetics for Technical Products and Innovation: An Overview for Applications.

[51] Ahijado Aparicio P. (2016). The Team Creative Process in Biomimetics: A Transactive Memory Theory Perspective. Master’s Thesis.

[52] Eggermont M.J. (2018). Bio-Inspired Design and Information Visualization.

[53] Helms M. 16 Challenges for BID in Industry. Proceedings of the NASA VINE Tools Workshop.

[54] Jacobs S., Eggermont M., Helms M., Wanieck K. (2022). The Education Pipeline of Biomimetics and Its Challenges. Biomimetics.

[55] Stevens L.L., Fehler M., Bidwell D., Singhal A., Baumeister D. (2022). Building from the Bottom Up: A Closer Look into the Teaching and Learning of Life’s Principles in Biomimicry Design Thinking Courses. Biomimetics.

[56] McInerney S.J., Niewiarowski P.H. (2022). Biomimicry Training to Promote Employee Engagement in Sustainability. Biomimetics.

